# Supplementary material for: Integrative analysis of mutated genes and mutational processes reveals novel mutational biomarkers in colorectal cancer
Source: BMC Bioinformatics. 2022 Apr 19;23:138. doi: 10.1186/s12859-022-04652-8 (PMC9017053; doi:10.1186/s12859-022-04652-8)

Figure S1. Mutation rates in the CRC patients

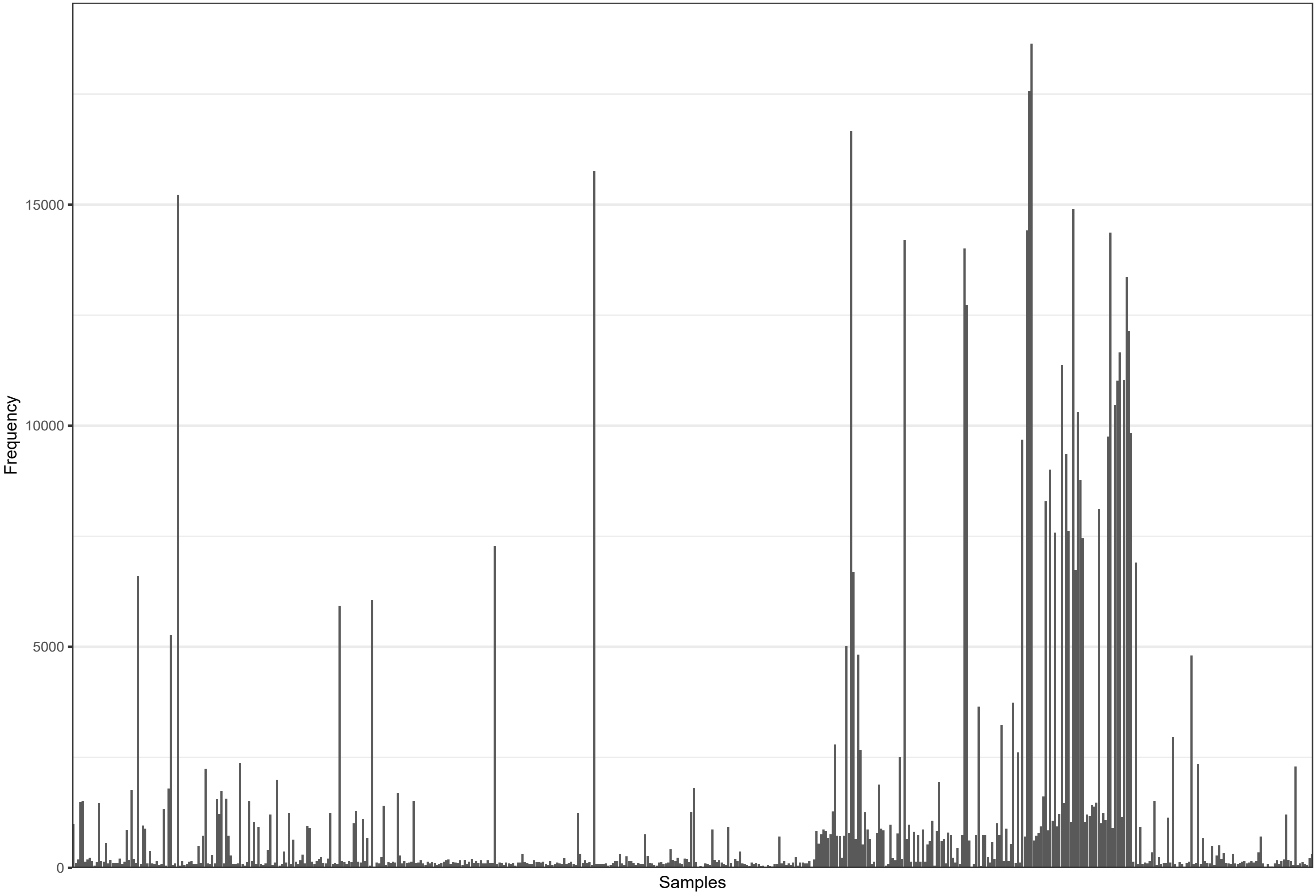

Figure S2. Identify best-fitted distribution to identify significant genes

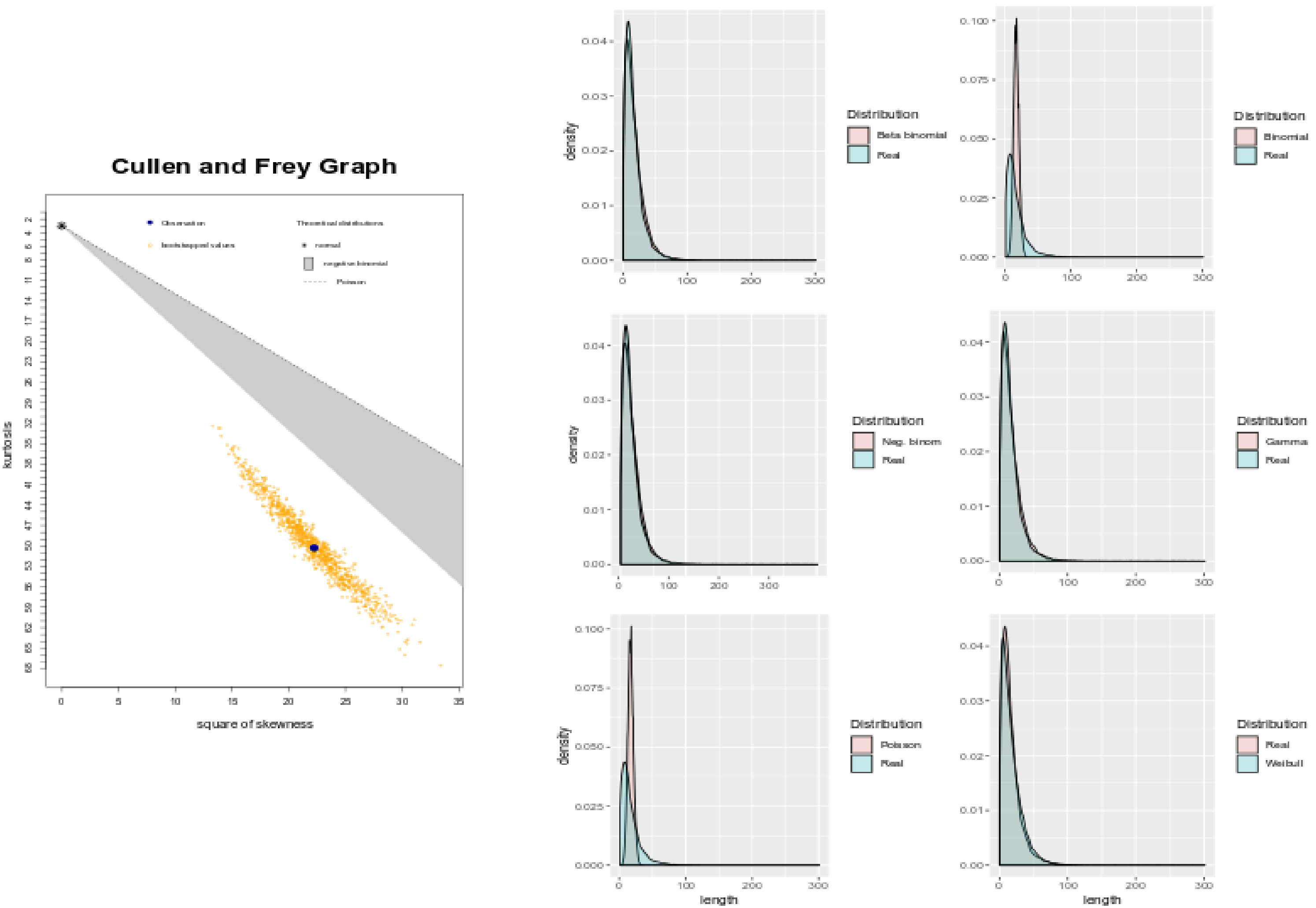

Figure S3. Identify best-fitted distribution to identify significant gene-motifs

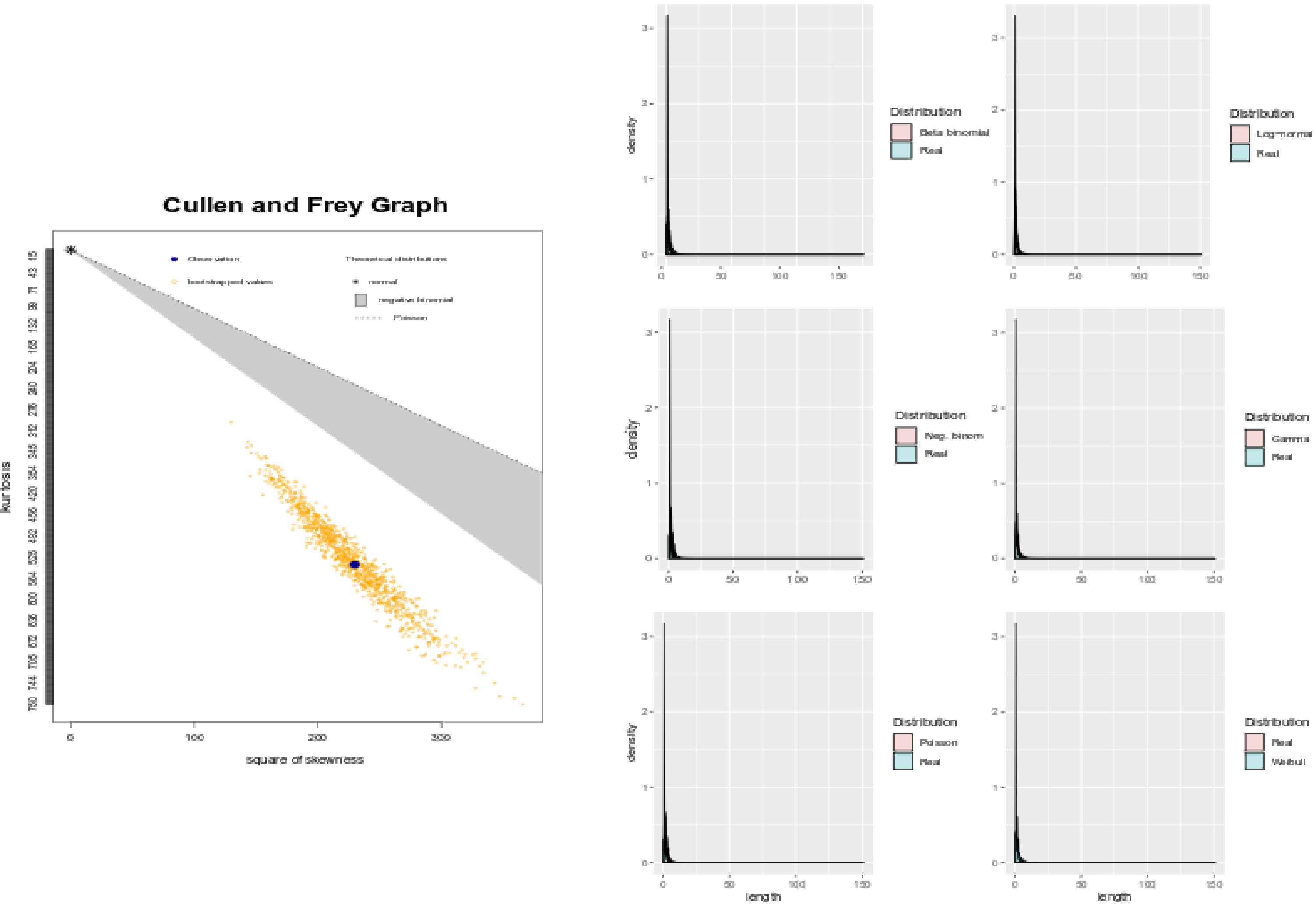

Figure S4. An overview of gene-motif concept

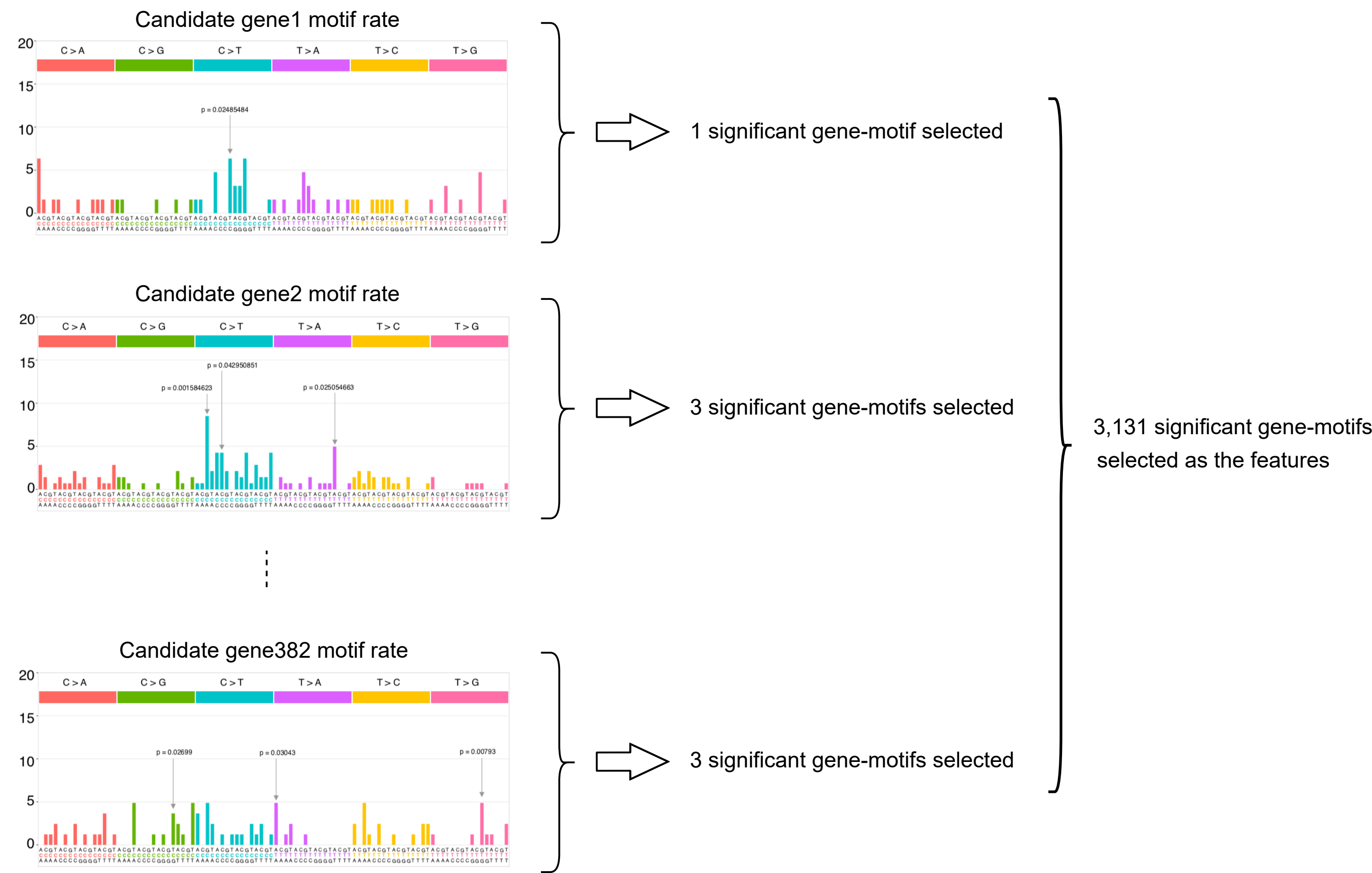

Figure S5. Selected features in two most significant PCAs before scaling

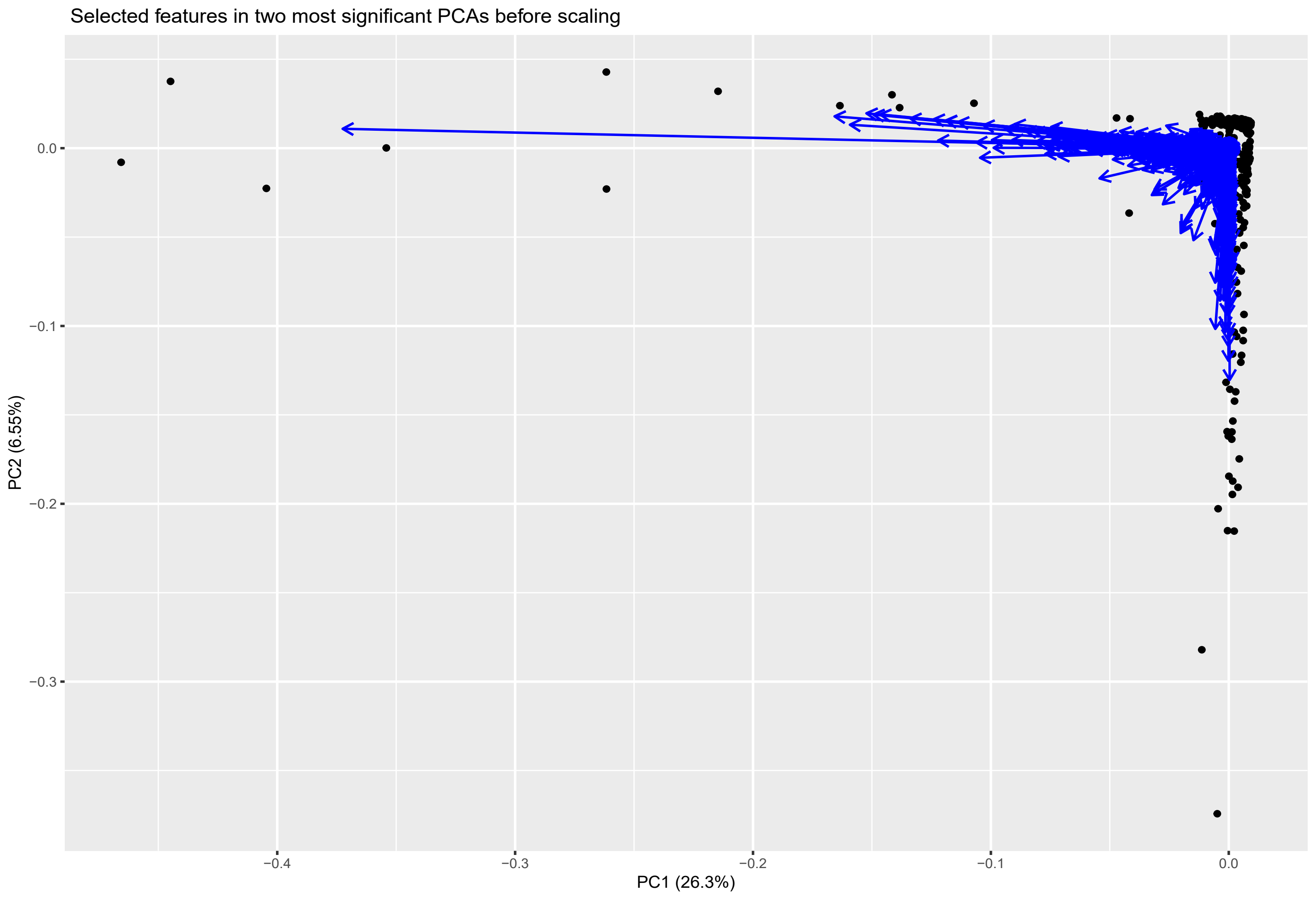

Figure S6. Illustration of patients in two first PCAs of features

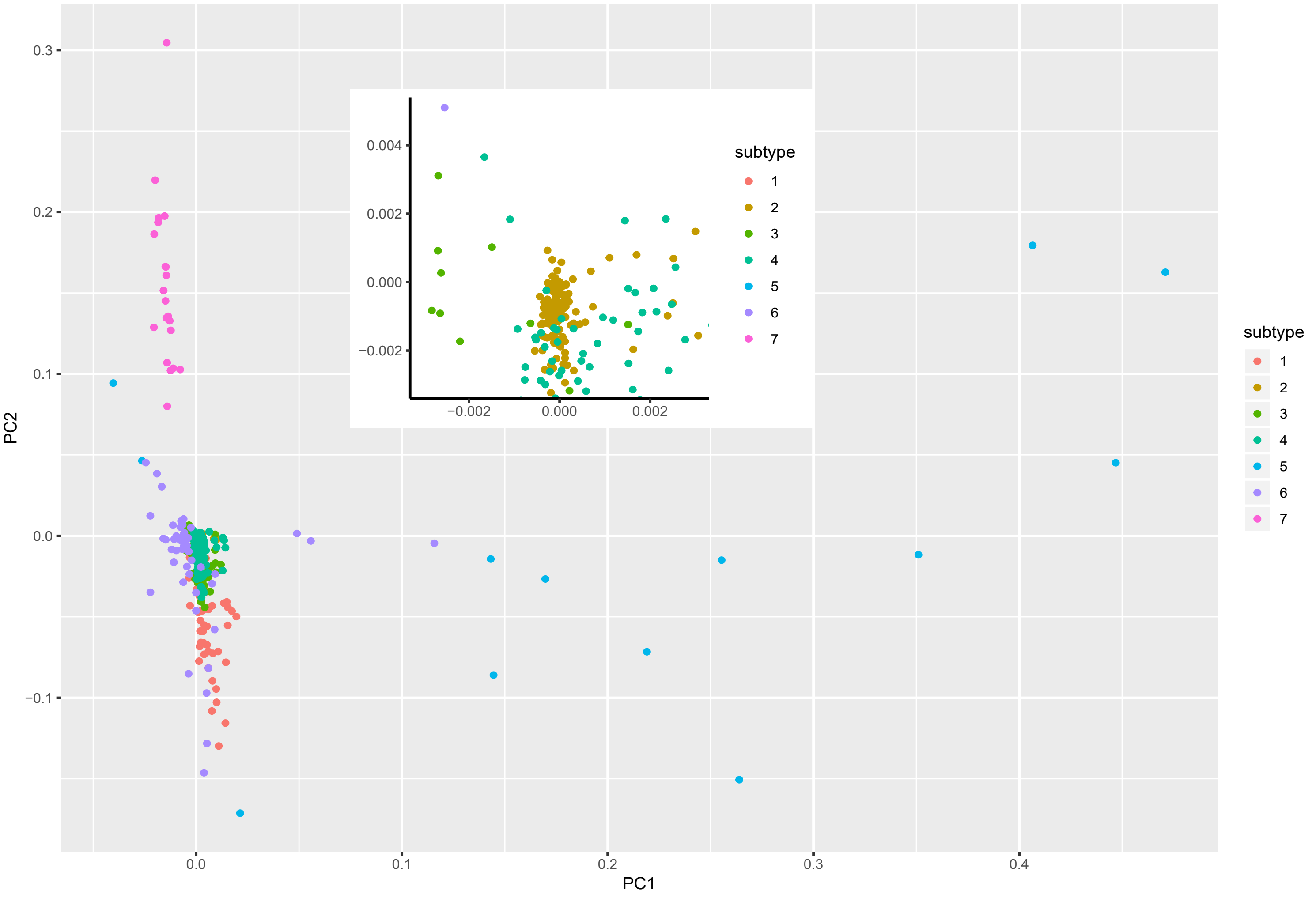

Figure S7. Correlation between our identified signatures and Alexandrov's signatures

	Alexandrov1	Alexandrov2	Alexandrov3	Alexandrov4	Alexandrov5	Alexandrov6	Alexandrov7	Alexandrov8	Alexandrov9	Alexandrov10	Alexandrov11	Alexandrov12	Alexandrov13	Alexandrov14	Alexandrov15
signature1	0.31	0.28	0.08	0.09	0.20	0.33	0.33	0.16	0.16	0.39	0.31	0.11	0.28	0.31	0.30
signature2	0.37	0.30	0.07	0.14	0.28	0.42	0.22	0.25	0.22	0.38	0.36	0.12	0.22	0.40	0.37
signature3	0.31	0.28	0.26	0.25	0.30	0.27	0.24	0.30	0.35	0.46	0.16	0.19	0.31	0.34	0.30
signature4	0.78	0.72	0.74	0.56	0.80	0.66	0.65	0.76	0.81	0.66	0.62	0.75	0.64	0.62	0.68
signature5	0.60	0.47	0.40	0.38	0.65	0.68	0.44	0.41	0.58	0.54	0.50	0.73	0.39	0.55	0.65
signature6	0.52	0.42	0.27	0.35	0.45	0.63	0.34	0.35	0.34	0.49	0.57	0.34	0.41	0.64	0.58
signature7	0.72	0.74	0.60	0.67	0.67	0.65	0.69	0.59	0.50	0.54	0.79	0.53	0.63	0.67	0.65

	Alexandrov16	Alexandrov17	Alexandrov18	Alexandrov19	Alexandrov20	Alexandrov21	Alexandrov22	Alexandrov23	Alexandrov24	Alexandrov25	Alexandrov26	Alexandrov27	Alexandrov28	Alexandrov29	Alexandrov30
signature1	0.12	0.25	0.26	0.27	0.27	0.24	0.16	0.31	0.22	0.23	0.25	0.24	0.15	0.05	0.06
signature2	0.08	0.30	0.32	0.37	0.32	0.30	0.26	0.38	0.22	0.27	0.31	0.31	0.12	0.10	0.10
signature3	0.28	0.29	0.30	0.24	0.25	0.13	0.20	0.18	0.27	0.28	0.23	0.26	0.31	0.08	0.10
signature4	0.80	0.76	0.65	0.73	0.70	0.66	0.61	0.59	0.55	0.75	0.74	0.70	0.49	0.43	0.35
signature5	0.63	0.57	0.43	0.55	0.67	0.75	0.45	0.49	0.33	0.53	0.72	0.51	0.15	0.22	0.11
signature6	0.27	0.32	0.43	0.56	0.51	0.42	0.38	0.57	0.43	0.37	0.46	0.48	0.20	0.12	0.12
signature7	0.57	0.61	0.71	0.75	0.66	0.54	0.54	0.77	0.65	0.57	0.61	0.71	0.24	0.26	0.33

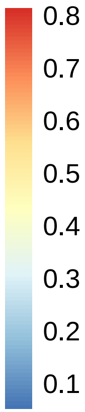

Figure S8. Mutational load in coding genes

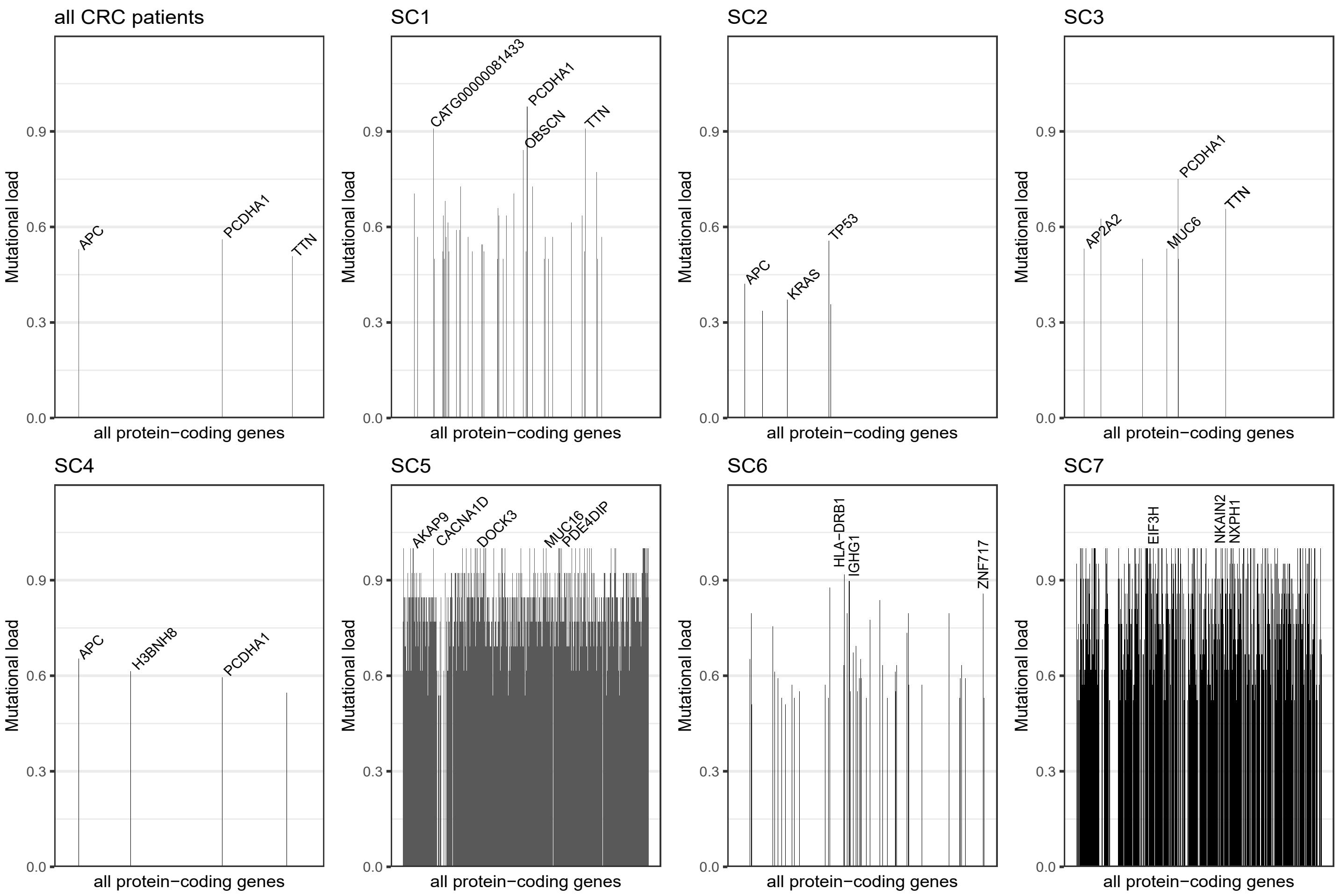

Figure S9. Mutational load in long non-coding genes

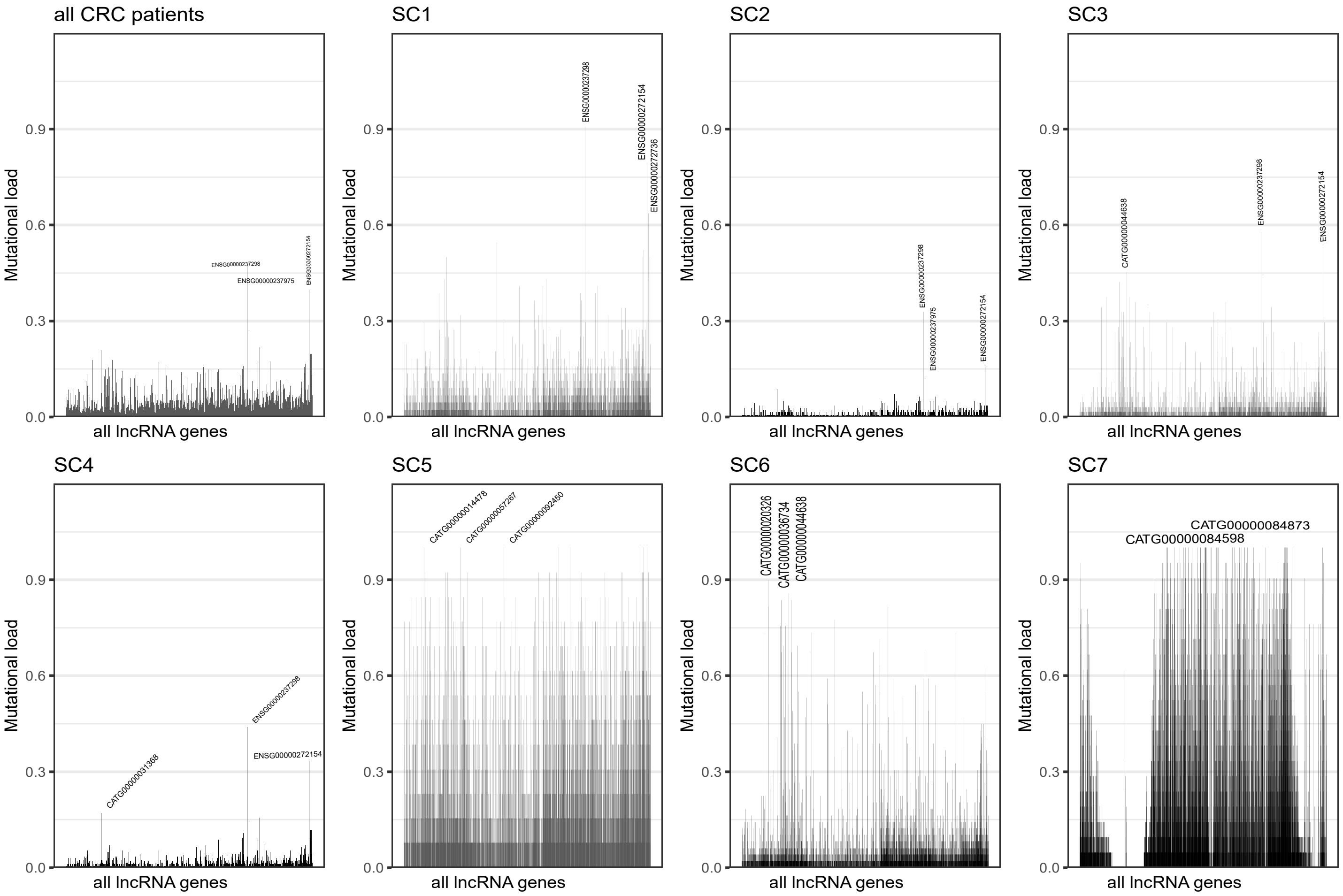

Figure S10. Mutation rates in coding and lncRNA genes in each subtype

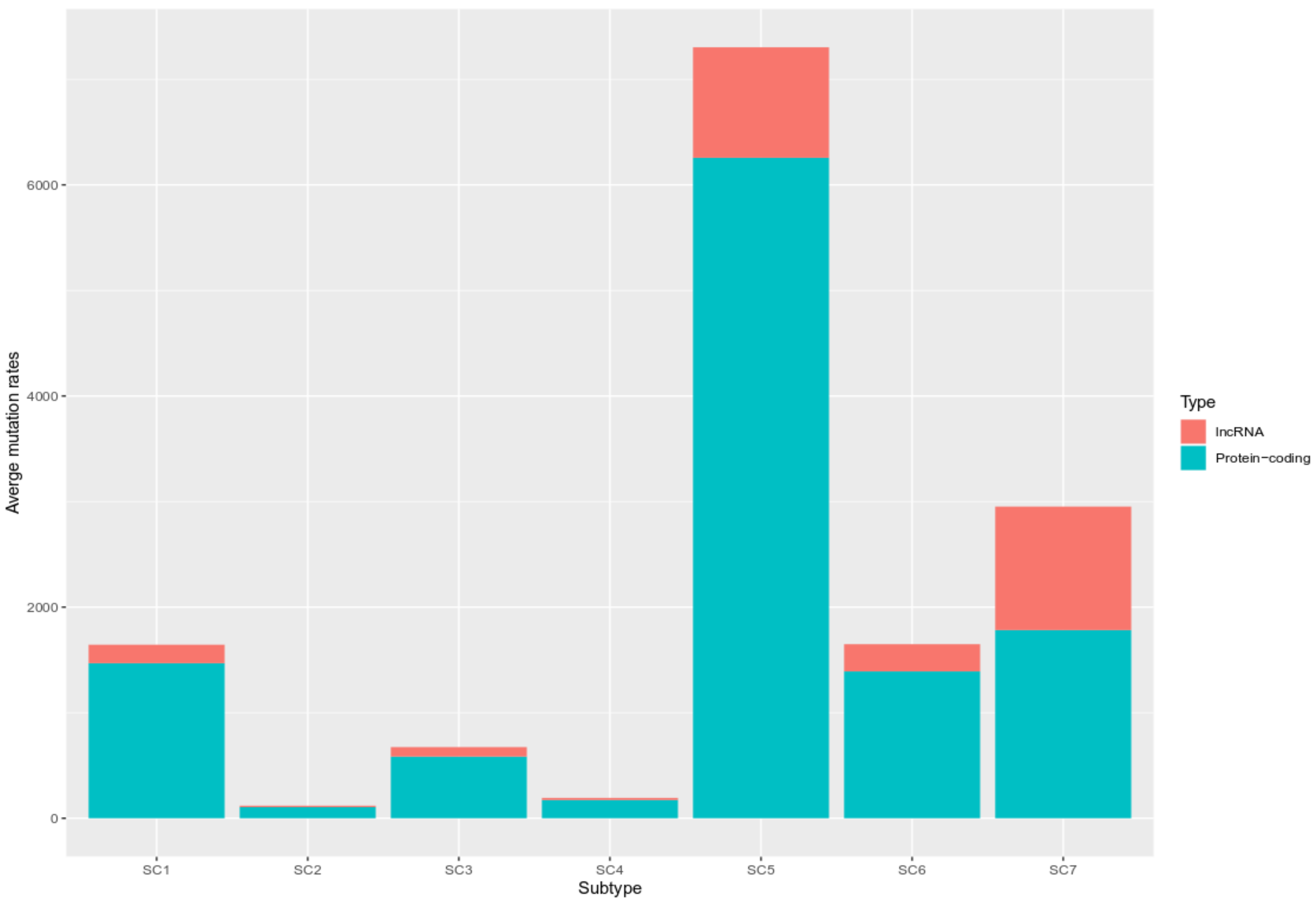

Figure S11. Consequence type analysis

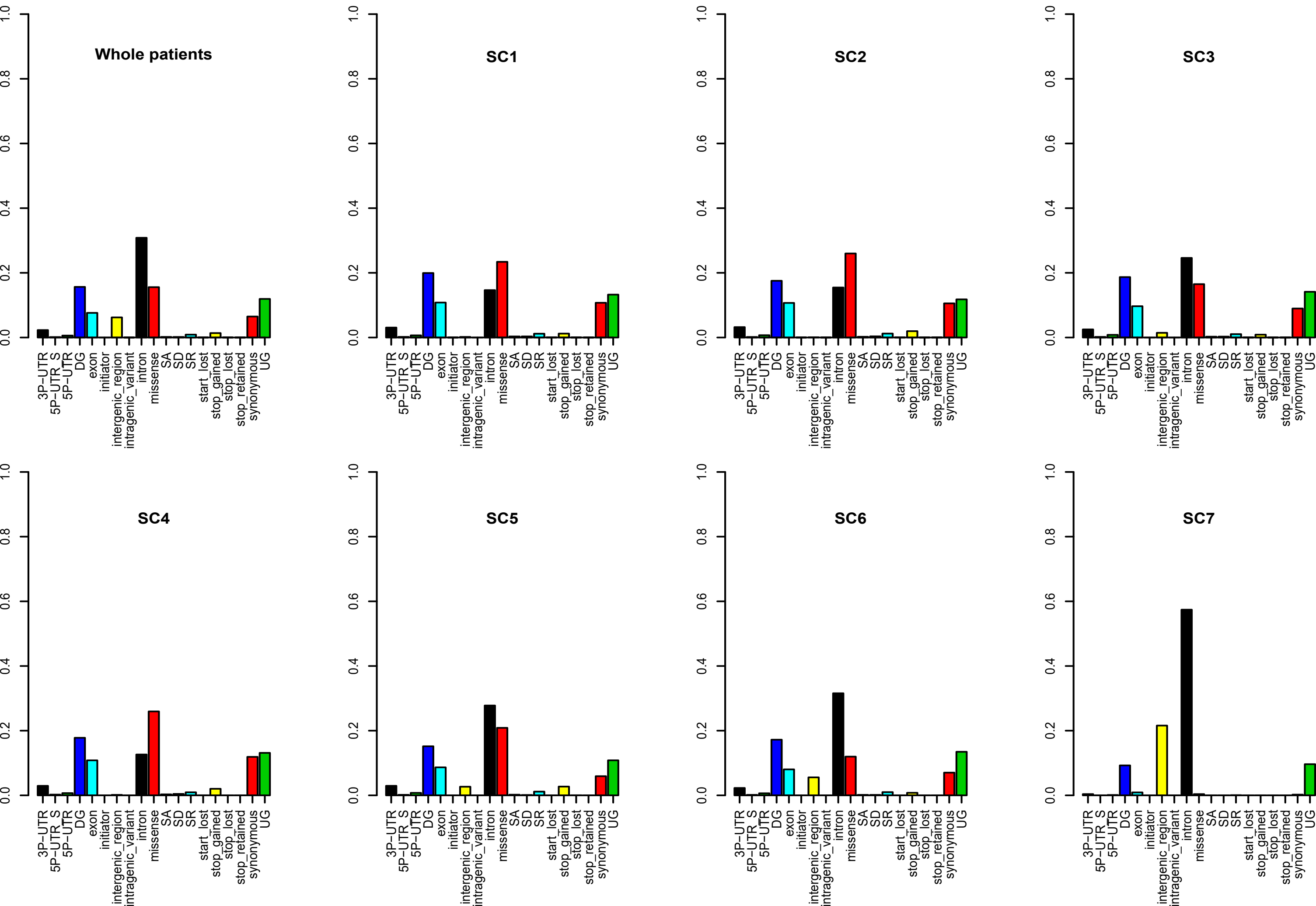

Figure S12. Mutational rate in difference transcripts of genes

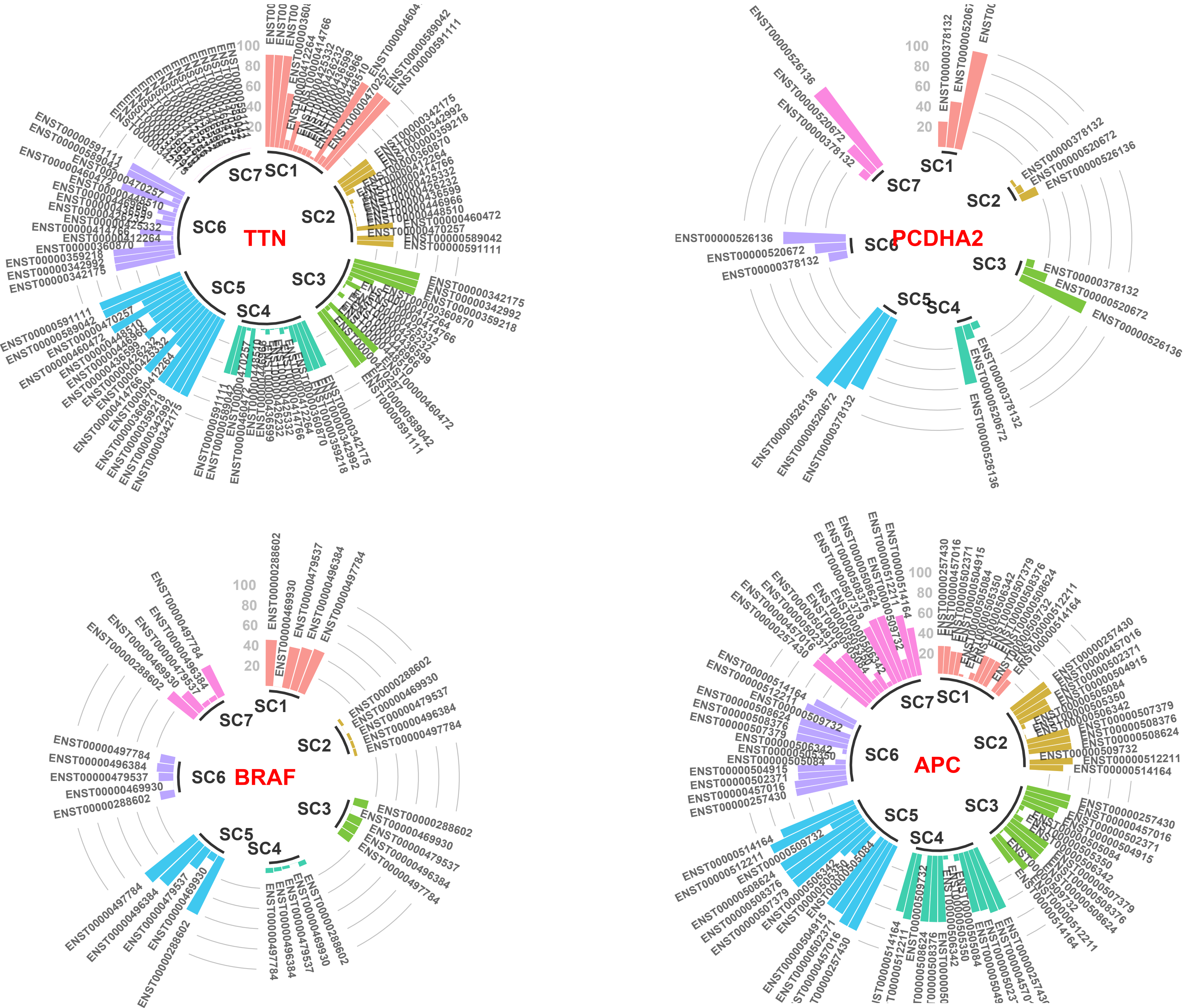

Figure S13. Analysis of age distribution of samples in the identified subtypes

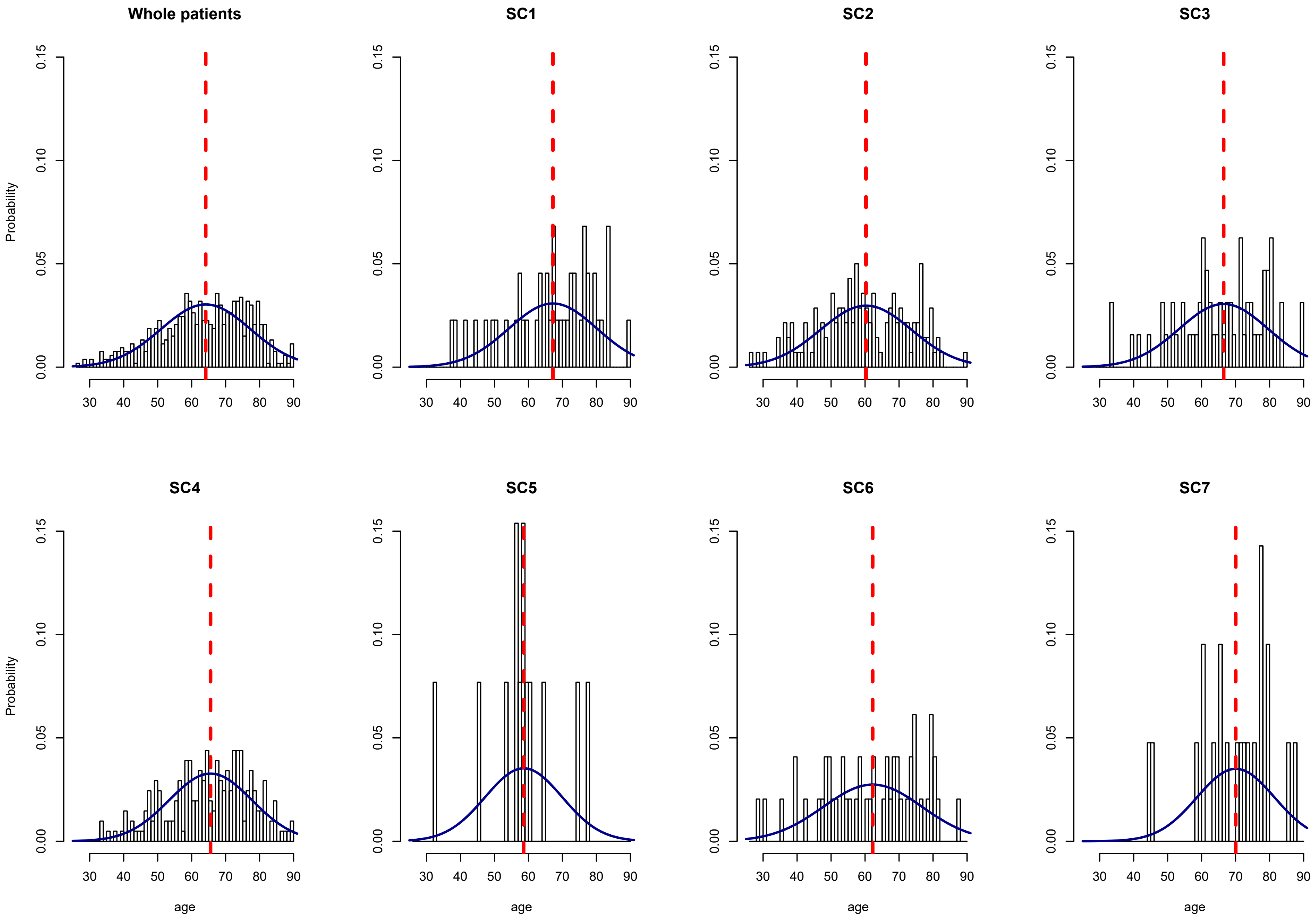

Figure S14. Evaluation diagram for mutational signature analysis

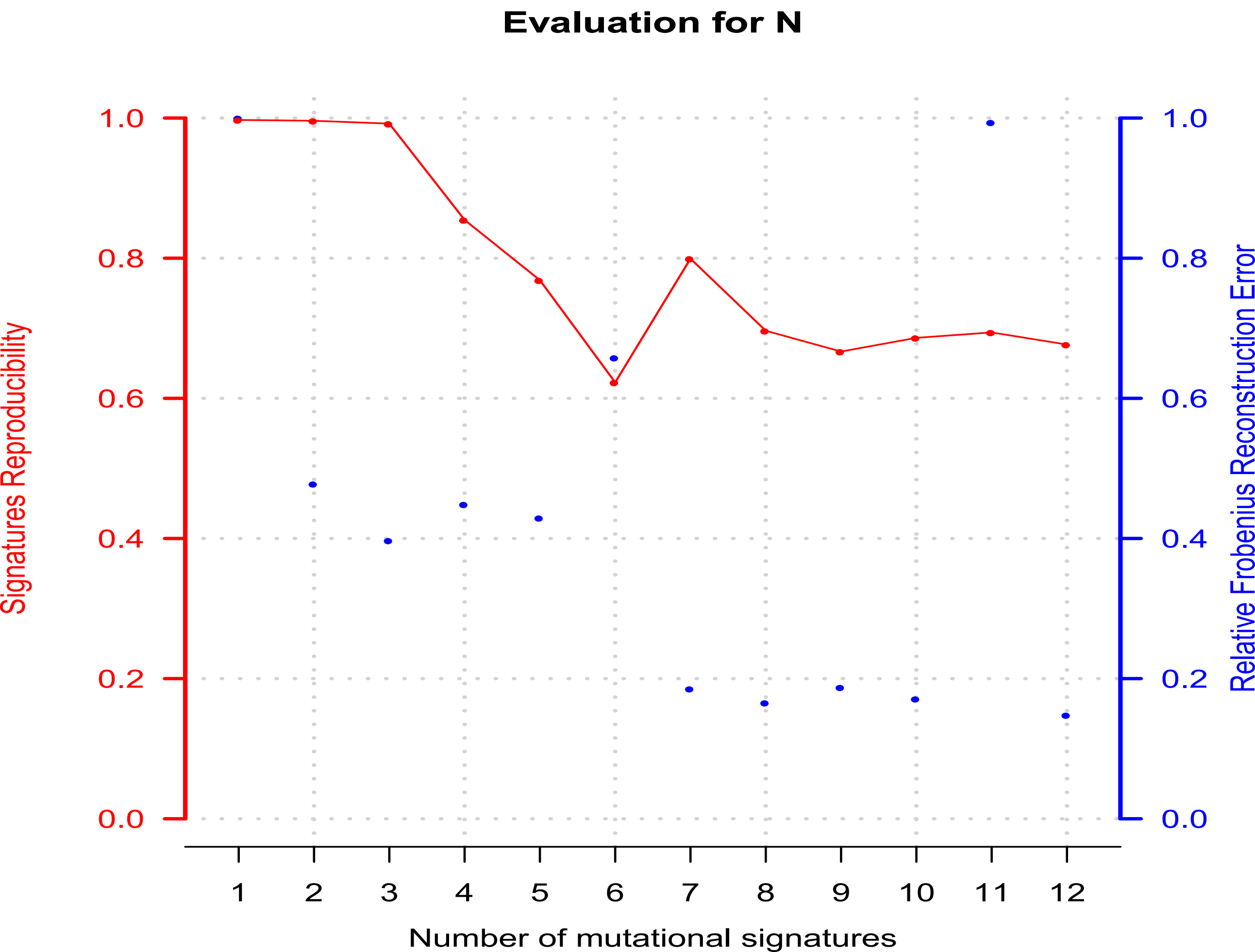

Supplement: Supplementary file 2 — Additional file 2: Figure S1. Mutation rates of the CRC patients. This plot shows number of mutations in each CRC sample. Figure S2. Identify best-fitted distribution to discover significant genes. This plot shows our comparison of different distribution techniques to fit the number of mutations in the genes and identify significantly mutated genes. Figure S3. Identify best-fitted distribution to discover significant gene-motifs. This plot shows our comparison of different distribution techniques to fit the number of mutations in the gene-motifs and identify significantly mutated gene-motifs. Figure S4. An overview of gene-motifs concept. We first identify 382 significantly mutated coding genes in colorectal cancers (candidate genes). We then used Fisher exact test to identify those motifs that significantly mutated within candidate genes. Figure S5. Selected 3131 features in two most significant PCAs before scaling. This plot shows two principal components (PCs) that demonstrates the potential discrimination that can be obtained from our identified features. Figure S6. Illustration of patients in two first PCAs of features. Distribution of CRC samples through 3131 gene-motif features by PCA analysis. Figure S7. Correlation between our identified signatures and Alexandrov's signatures in each CRC subtype separately. Figure S8. Mutational load of protein coding genes in each subtype separately. Each bar chart shows fraction of samples with mutation in a gene. Figure S9. Mutational load of long non-coding RNA genes in each subtype separately. Each bar chart shows fraction of samples with mutation in a lncRNA. Figure S10. Mutation rates in coding and lncRNA genes in each subtype. Red color indicates average number of mutations in lncRNA genes and green color indicates average number of mutations in coding genes. Figure S11. Consequence type analysis. Figure shows fraction of mutations in different consequence types for each subtype. Figure S12. Mutation rate in transcr [file 12859_2022_4652_MOESM2_ESM.pdf]
